# Cuproptosis-related lncRNAs emerge as a novel signature for predicting prognosis in prostate carcinoma and functional experimental validation

**DOI:** 10.3389/fimmu.2024.1471198

**Published:** 2024-10-28

**Authors:** Yangbai- Lu, Jinfeng- Wu, Xianzhe Li, Qu- Leng, Jian- Tan, Hongxing- Huang, Rui- Zhong, Zhenjie- Chen, Yongxin- Zhang

**Affiliations:** ^1^ Department of Urology, Zhongshan City People’s Hospital, Zhongshan, Guangdong, China; ^2^ Department of First Clinical Medical College, Guangdong Medical University, Zhanjiang, Guangdong, China; ^3^ Division of Clinical Epidemiology and Aging Research, German Cancer Research Center (DKFZ), Heidelberg, Germany; ^4^ Medical Faculty Heidelberg, Heidelberg University, Heidelberg, Germany; ^5^ Department of MR, Zhongshan City People’s Hospital, Zhongshan, Guangdong, China

**Keywords:** cuproptosis, lncRNAs, prognosis signature, prostate carcinoma, SNHG9

## Abstract

**Background:**

Prostate cancer (PCa) is one of the most common malignancies of the urinary system. Cuproptosis, a newly discovered form of cell death. The relationship between cuproptosis-related long non-coding RNAs (ClncRNAs) related to PCa and prognosis remains unclear. This study aimed to explore the clinical significance of novel ClncRNAs in the prognostic assessment of PCa.

**Methods:**

ClncRNAs and differentially expressed mRNAs linked to these ClncRNAs were identified using Pearson’s correlation and differential expression analyses. A prognostic signature (risk score) comprising three ClncRNAs was established based on multivariable Cox regression analysis. The predictive performance of this ClncRNAs signature was validated using receiver operating characteristic curves and nomograms. Finally, further *in vitro* cell experiments were conducted for validation, including quantitative polymerase chain reaction (qPCR), western blot (WB), cell proliferation assays, cell migration assays, cell invasion assays, apoptosis, and cell cycle analysis.

**Results:**

We constructed a prognostic signature of ClncRNAs for PCa comprising three key differentially expressed ClncRNAs(AC010896-1, AC016394-2, and SNHG9). Multivariable Cox regression analysis indicated that clinical staging and risk scores of the ClncRNAs signature were independent prognostic factors for PCa. Compared to other clinical features, the ClncRNAs signature exhibited higher diagnostic efficiency and performed well in predicting the 1-, 3-, and 5-year progression-free intervals (PFIs) for PCa. Notably, in terms of immune activity, PCa patients with high-risk scores exhibited higher tumor mutational burden (TMB) levels, while their Tumor Immune Dysfunction and Exclusion (TIDE) scores were lower than those of PCa patients with low-risk scores. Additionally, *in vitro* cellular functional experiments, we knocked down SNHG9 that is the most significantly differentially expressed ClncRNA among the three key ClncRNAs. SNHG9 knockdown resulted in a significant increase in G1 phase cells and a decrease in S and G2 phases, indicating inhibition of DNA synthesis and cell cycle progression. Colony formation assays showed reduced clonogenic ability, with fewer and smaller colonies. Western blot analysis revealed the upregulation of the key cuproptosis-related mRNAs FDX1 and DLST. These findings suggested that SNHG9 promotes PCa cell proliferation, migration, and invasion.

**Conclusion:**

Building on the three ClncRNAs, we identified a novel prognostic signature of PCa. The ClncRNA SNHG9 can promote PCa cell proliferation, migration, and invasion.

## 1 Introduction

Estimates suggest that globally, there are close to 1.5 million new cases of prostate cancer (PCa), resulting in approximately 397,000 deaths. In 2022, it was the second most prevalent cancer among men and the fifth leading cause of cancer-related deaths among men (1). Although surgery, hormonal therapy, and radiation therapy show significant therapeutic efficacy in most PCa cases (2), the heterogeneity of PCa often renders these treatments ineffective. Therefore, novel prognostic methods and treatment strategies for PCa are urgently needed.

Biological features, primarily presented through gene expression and genomic profiling, can enhance the predictive capacity of traditional clinicopathological features (3). Understanding the genes associated with PCa may improve treatment selection and precision. Cuproptosis is a novel cell death mechanism that is distinct from other known mechanisms such as apoptosis, autophagy, and ferroptosis. Copper ions can induce cell death even when known cell death pathways are blocked. During mitochondrial respiration, copper ions directly bind to lipidated components of the tricarboxylic acid cycle, leading to protein aggregation. Additionally, copper ions can decrease the protein levels of Fe-S clusters, triggering proteinotoxic stress responses, and ultimately leading to cell death (4). By leveraging this novel approach to cell death, we are developing new strategies to advance the development of novel therapeutic options for patients with late-stage PCa.

Within the human genome, only a minority of genes encode proteins, with the majority encoding non-coding RNAs. Long non-coding RNAs (lncRNAs) are single-stranded RNAs that are more than 200 nucleotides long and lack protein-coding capabilities (5). lncRNAs regulate various physiological and biochemical cellular processes through chromatin modification, transcriptional activation, and interference (6, 7). lncRNAs can serve as non-invasive tumor markers for malignancies of the genitourinary system. Further elucidation of the molecular mechanisms by which lncRNAs function in normal and malignant cells will enhance our understanding of tumor biology and provide new therapeutic targets for genitourinary cancers (8). Recent studies have increasingly confirmed the crucial role of cuproptosis-related lncRNAs (ClncRNAs) in the prognosis and immunity of various genitourinary tumors. Zhang et al. (9) identified the ClncRNAs LINC01711 as a potential biomarker for early diagnosis and prognosis of clear cell renal carcinoma. Shen et al. (10) developed a risk signature comprising eight ClncRNAs with potential clinical applications in predicting outcomes and diagnosing and treating bladder cancer. However, research on the relationship between ClncRNAs and the prognosis of patients with PCa remains limited. Therefore, this study aimed to construct a novel prognostic signature related to ClncRNAs to predict the prognosis of PCa.

## 2 Materials and methods

### 2.1 Data download and pre−processing

Cuproptosis-related genes expression was standardized using the “limma” R package. Subsequently, the Perl programming language was used to distinguish between mRNAs and lncRNAs. Additionally, simple nucleotide variation data and masked somatic mutation data were downloaded from The Cancer Genome Atlas (TCGA) database and employed to compute the mutation burden of PCa. Nineteen cuproptosis-related mRNAs were sourced from previously published studies including NFE2L2, NLRP3, ATP7A, ATP7B, SLC31A1, FDX1, LIAS, LIPT1, LIPT2, DLD, DLAT, PDHA1, PDHB, MTF1, GLS, CDKN2A, DBT, GCSH, and DLST (4).

### 2.2 Screening the differentially expressed ClncRNAs

Pearson correlation analysis was employed to explore the association between ClncRNAs and PCa-related lncRNAs, using a screening criterion of |R²| >0.5 and p <0.05 to ensure the significance and robustness of the discovered correlations. Subsequently, further analysis was conducted using the “limma” R package, adjusting for p <0.05 and |log2 fold change (FC)| >1 as criteria to screen for differentially expressed ClncRNAs. These ClncRNAs then were visualized using Sankey plots and volcano plots to illustrate the aforementioned results.

### 2.3 Establishment and evaluation of a novel prognostic signature associated with ClncRNAs

Six candidate ClncRNAs, including two protective and four risk lncRNAs, were identified using univariate Cox regression analysis. Subsequently, to prevent overfitting, LASSO regression analysis and lambda spectra were employed to explore collinearity. Next, three key ClncRNAs were identified by multivariable Cox regression analysis. Finally, a prognostic ClncRNAs signature based on these three ClncRNAs was constructed and 420 patients were divided into high- and low-risk groups using the median score as the cutoff value. In this study, the risk score calculation formula of ClncRNAs signature was as follows: risk score = $f\left(x\right)=\sum_{i=1}^{n}\left({\text{Coef}}_{i}{*\text{Exp}}_{\text{i}}\right)$ , where Coef*_i_* represents the corresponding coefficient for each ClncRNAs and Exp_i_ represents the gene expression level of the selected ClncRNAs. Subsequently, the Kaplan–Meier (K–M) method was used to plot the progression-free interval (PFI) and disease-free interval (DFI) of patients with PCa using the “survival” R package. Based on the risk score and clinical features, time-dependent receiver operating characteristic (ROC) curves and C-index curves were plotted using the “timeROC” R package to evaluate the accuracy and stability of the signature. Additionally, based on the results of multivariable Cox regression analysis, a nomogram was developed using the “rms” package to predict the 1-, 3-, and 5-year PFI and evaluate the long-term predictive accuracy of the signature. Furthermore, the correlation between the ClncRNAs signature and some clinical features was assessed, and their predictive ability in subgroups with different clinical features was evaluated using KM survival analysis.

### 2.4 Principal components analysis and functional enrichments analysis

Following the identification of differentially expressed mRNAs between the high-risk and low-risk groups, we conducted gene ontology (GO) and Kyoto Encyclopedia of Genes and Genomes (KEGG) pathway analyses using the “clusterProfiler” and “enrichment” packages to elucidate molecular functions and key signaling pathways (11, 12). The components of graphene oxide comprised biological process components, cellular component components, and molecular function components.

### 2.5 Calculation of tumor mutation burden score

The tumor mutational burden (TMB) reflects the number of mutations in a tumor. Mutation data from PCa samples were downloaded from TCGA, and analysis was conducted using the R package “maftools” (13). The TMB is correlated with the clinical efficacy of immunotherapy. Additionally, we explored the differences in somatic mutation characteristics between the high-risk and low-risk groups.

### 2.6 A comparative analysis of immune cell infiltration and immune-related functions in different risk groups

To evaluate the differences in immune infiltration between high-risk and low-risk groups, we performed Gene Set Variation Analysis (GSVA) using the “reshape2” and “GSEABase” packages and generated heatmaps to illustrate the variations in immune functions across different risk groups (14). Additionally, we obtained Tumor Immune Dysfunction and Exclusion (TIDE) scores from their website (http://tide.dfci.harvard.edu) and analyzed the differences in TIDE scores between the low-risk and high-risk groups. Subsequently, we examined other immune scores, including T cell dysfunction, T cell exclusion, PD-L1, CD8, IFNG, Merck18, CAF, TAM M2, and MDSC scores, to assess the differences in immune infiltration between the high-risk and low-risk groups. These scores are sourced from the TIDE website.

### 2.7 Cell lines, cell culture, and handling

The PCa cell lines RWPE-1, PC-3, U145, 22RV1, LNCaP, C4-2PC3, and DU145 were purchased from the ATCC (Manassas, VA, USA). PC-3 and DU145 cells were transfected using Lipofectamine™ RNAiMAX (Invitrogen, Cat. No. 13778075) using LncRNA-SNHG9-targeting small interfering RNA (siRNA) designed and synthesized by Beijing TsingKe Biological Technology Co., Ltd. The sequences for siSNHG9-1, siSNHG9-2, and siSNHG9-3 were as follows: siSNHG9-1 sequence: ACCCGAAGAGUGGCUAUAATT, siSNHG9-2 sequence: CCUCUUCACUUAGGACACUTT, and siSNHG9-3 sequence: CCACGUCUUUCAAAUAAAGTT. PC-3 and DU145 cells were cultured in the RPMI-1640 (Gibco, Cat. No. C11875500BT) and MEM (Gibco, Cat. No. C11095500BT) media, respectively, supplemented with 10% fetal bovine serum (FBS) and 1% dual antibiotics (penicillin-streptomycin) (HyClone, Cat. No. SH30010). Cultures were maintained at 37°C in a humidified atmosphere containing 5% CO_2_.

### 2.8 RNA Extraction, reverse transcription, and quantitative real-time polymerase chain reaction

Total RNA was extracted from RWPE-1, PC-3, U145, 22RV1, LNCaP, C4-2PC3, and DU145 cells using the TRIzol reagent. The concentration and quality of each RNA sample were evaluated by measuring the OD values using a BioPhotometer Plus (Eppendorf Nucleic Acid and Protein Analyzer). Subsequently, the extracted RNA was reverse transcribed into complementary DNA (cDNA) using the EasyScript First-Strand cDNA Synthesis SuperMix Kit. The resulting cDNA was subjected to quantitative real-time polymerase chain reaction (qRT-PCR) using SYBR Green Master Mix (Vazyme). Analysis was performed on the ABI PRISM^®^ 7500 Sequence Detection System, with GAPDH serving as the internal reference gene. Relative gene expression levels were calculated using the 2^-ΔΔCt^ method. PCR primers for the target gene (SNHG9) and the internal reference gene (GAPDH) are provided in **Supplementary Table 2**.

### 2.9 Cell proliferation experiment

The proliferative capacity of PC-3 and DU145 cells transfected with siRNA was assessed using the Cell Counting Kit-8 (CCK-8) reagent according to the manufacturer’s instructions. PC-3 and DU145 cells were seeded in 96-well plates, and CCK-8 solution (Beyotime, Cat. No. C0039) was added at 0, 24, 48, and 72 h. After incubation for 4 h, optical density (OD450) was measured using a microplate reader (Thermo Fisher Scientific, Multiscan MK3) to determine the absorbance values for CCK-8 detection.

### 2.10 Cell apoptosis assay

Apoptosis was examined using an Annexin V-FITC Apoptosis Detection Kit (Keygen, Cat. No. KGA106) using flow cytometry. All procedures were performed according to manufacturer’s instructions. Briefly, cells pretreated with phosphate-buffered saline (PBS) were washed twice to obtain a pellet. Subsequently, the cells at different stages of apoptosis were identified using fluorescein isothiocyanate and propidium iodide (PI) solutions. The cells were then incubated in the dark at room temperature (18–24°C) for 15 min. Finally, the apoptosis rate was measured using a BD FACS Calibur flow cytometer (Beckman Coulter, CA, USA).

### 2.11 Flow cytometric cell cycle analysis

A KGA511 cell cycle assay kit was used to assess the cellular proliferation status and cycle distribution under various conditions. Following transfection for 48 h, 1 × 10^6^ cells were collected per sample, centrifuged to remove the supernatant, and washed twice with pre-chilled PBS. Subsequently, cells were treated with pre-chilled 70% ethanol and fixed overnight at 4°C or stored long-term at -20°C. Fixed cells were then collected by centrifugation, washed once with 1 mL of PBS, and resuspended in PBS solution containing 50 μg/mL PI, 100 μg/mL RNase A, and 0.2% Triton X-100. Following 30 min of incubation at 4°C in the dark to stain the cellular DNA, an appropriate volume of the stained cell suspension was subjected to standard flow cytometry analysis. The results were analyzed using the ModFit cell cycle analysis software, which allocates cells to different cell cycle phases based on DNA content and generates the corresponding cell cycle distribution plots.

### 2.12 Transwell assay for assessing cell migration and invasion capacity

Transwell cell culture inserts (BD, REF353097) were used for the transwell migration assays. Approximately 1 × 10^5^ cells were seeded in 100 µL of serum-free culture medium in the upper chamber, while 600 µL of complete culture medium was added to the lower chamber. Following incubation at 37°C with 5% CO_2_ for 12–48 h, the inserts were retrieved. The cells on the upper surface of the insert were gently removed using a cotton swab and fixed in 4% paraformaldehyde for 20 min. After a single wash with PBS, the cells were stained with crystal violet for 10 min, washed again with PBS, and subjected to cell counting and image analysis using an inverted microscope (OLYMPUS CKX41, U-CTR30-2) and Image J 1.44 software. For the transwell invasion assay, Matrigel (BD, 356234) was dissolved overnight at 4°C and diluted to a 1:3 ratio with pre-chilled serum-free culture medium. Forty microliters of diluted Matrigel was added to the pre-chilled transwell inserts and allowed to gel at 37°C for 2 h. Excess liquid was removed from the inserts, and 100 µL of serum-free culture medium was added to the upper chamber, while 600 µL was added to the lower chamber. After overnight equilibration at 37°C, cells transfected with siRNA were seeded at a density of 1 × 10^5^ cells in 100 µL of serum-free DMEM-F12 or MEM medium in the upper chamber. Complete culture medium was added to the lower chamber, and the cells were incubated at 37°C with 5% CO_2_ for 24 or 48 h. Following incubation, the cells on the upper surface of the insert were removed using a cotton swab and the remaining cells were fixed with 4% paraformaldehyde for 15 min. After a single wash with PBS, the cells were stained with crystal violet for 10 min, washed again with PBS, and subjected to observation and statistical analysis.

### 2.13 Cloning formation experiment

Using pancreatic enzymes, cells from different groups were initially digested and resuspended in 1 mL culture medium to achieve an appropriate cell count range. The cells were subsequently dissociated into single-cell suspensions and diluted to a concentration of 1 × 10^4^ cells/ml. The diluted cells were then seeded into a 96-well plate, with 300 µL of suspension added to each well. After seeding, culture medium was added to each well to a final volume of 2000 µL to ensure sufficient growth medium for the cells. The plate was gently rocked horizontally and vertically to evenly distribute cells within each well. Cell growth was observed daily. When individual cells formed clustered clones, the culture medium was aspirated from each well. The wells were washed twice with PBS or saline, and then 200 µL of crystal violet staining solution was added to each well to ensure coverage of the well bottom, followed by a 20-min incubation period. The 96-well plate was rinsed with tap water, gently washed, and air-dried. Finally, the number of colonies was calculated and the stained 96-well plates were analyzed and scanned using an enzyme-linked immunospot (ELISPOT) AID iSpot system.

### 2.14 Protein extraction and western blotting

After washing the cells with PBS, protein extraction was performed using a radioimmunoprecipitation assay buffer containing 1% PMSF. The samples were then centrifuged at 14,000 rpm for 5 min at 4°C in appropriate centrifuge tubes. The protein concentration was determined using a BCA Protein Assay Kit (Thermo Scientific, USA). Total protein samples were separated on 15% or 10% SDS-PAGE gels and transferred onto polyvinylidene fluoride (PVDF) membranes. Membranes were blocked with TBST containing 5% skim milk powder. Antibodies against FDX1 (1:1000, 12592-1-AP, Proteintech, China), DLAT (1:2000, 13426-1-AP, Proteintech, China), and GAPDH (1:1000, KC-5G5, Shanghai Kangcheng Biotech, China) were incubated at 4°C. After overnight washing with TBST, the membranes were incubated with secondary antibodies at 37°C for 50 min to 3 h. The PVDF membranes were developed using ECL solution, followed by three washes and imaging.

### 2.15 Statistical analysis

Statistical analyses for visualization were performed using R software (version 4.2.0, https://www.r-project.org/). The Wilcoxon test was used to assess the differential expression of ClncRNAs and mRNAs, with significance set at p <0.05.

## 3 Results

### 3.1 Clinical data of patients and identification of ClncRNAs

**Figure 1:**
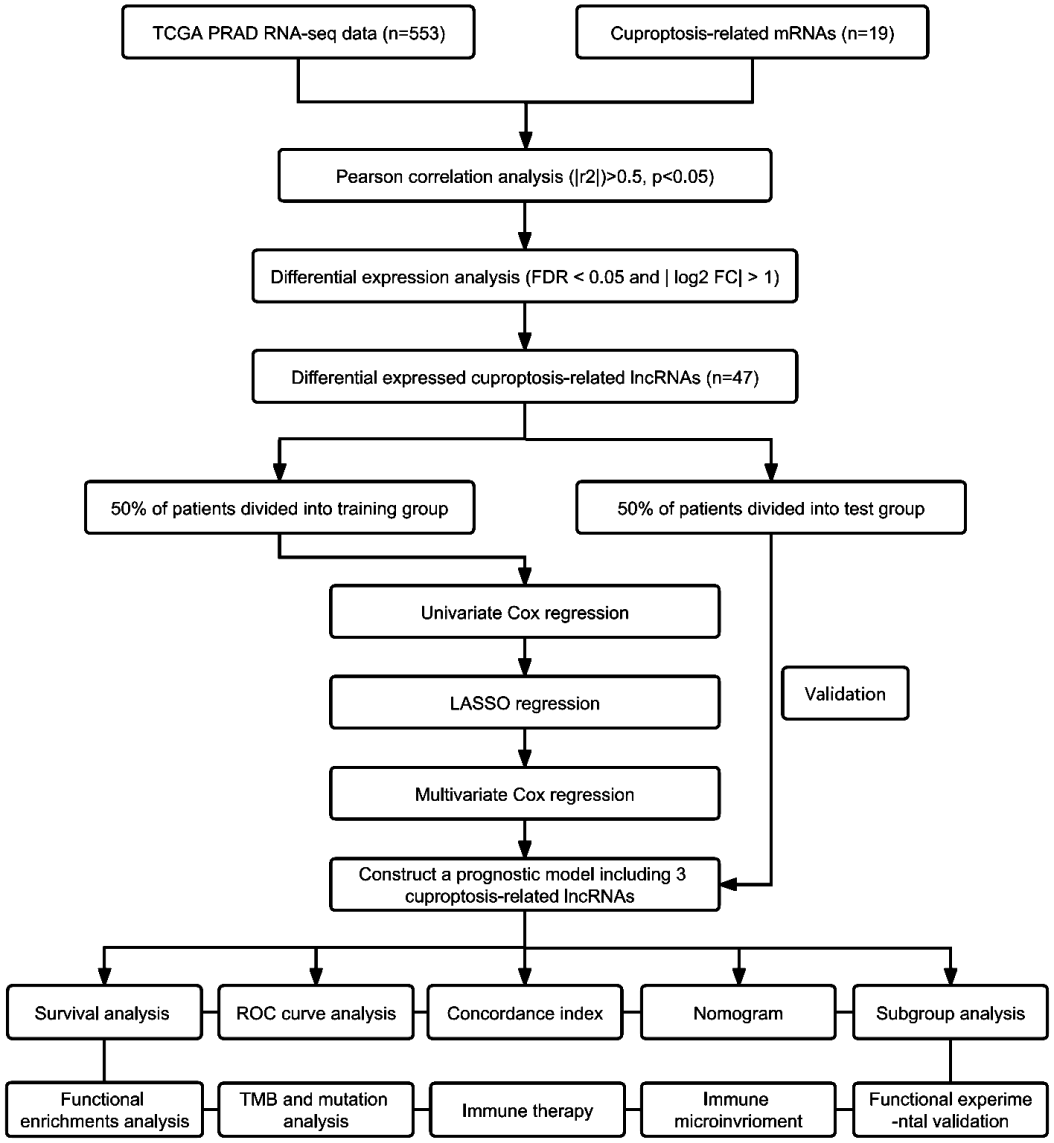
The schematic diagram of workflow.

**Figure 2:**
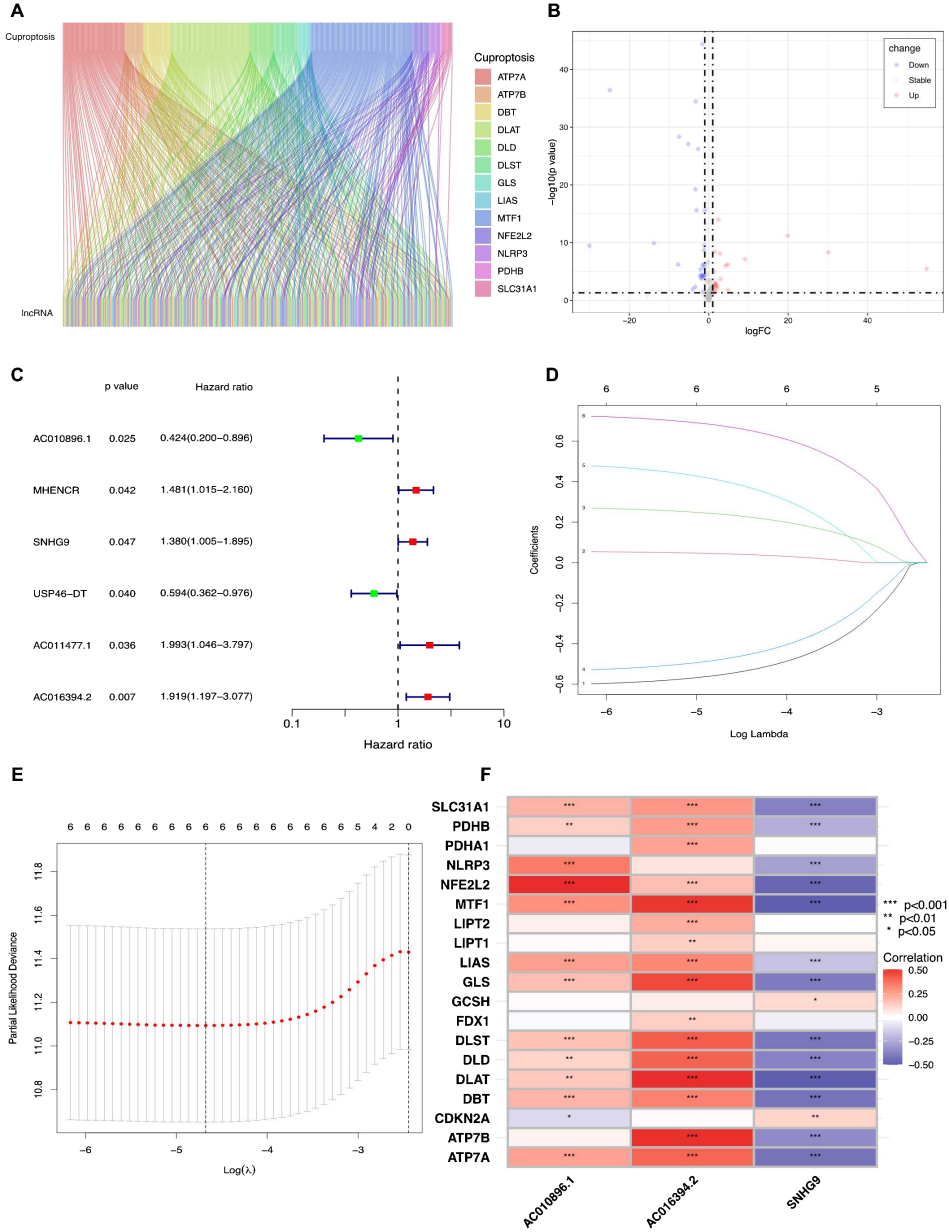
Identifying the ClncRNAs with prognostic significance in PCa. **(A)**The Sankey plot for the network of cuproptosis-related mRNAs and ClncRNAs. **(B)** The volcano diagram showed differentially expressed 47 ClncRNAs in PCa. **(C)** The forest plot displays six differentially expressed ClncRNAs with significant diagnostic value. **(D)** Lasso-Cox regression analysis is used to build a prognostic signature of ClncRNAs for PCa through 10-fold cross-validation for variable selection. **(E)** The LASSO penalty lambda (λ) of the six ClncRNAs. **(F)** The correlations of three prognostic ClncRNAs and cuproptosis-related mRNAs. *p <0.05, **p <0.01, and ***p <0.001.

The flowchart in **Figure 1** illustrates the process followed in this study. Gene expression profiles and clinical data of 52 normal and 501 PCa samples were obtained from the TCGA database. Detailed clinical characteristics of all the participants are presented in **Table 1**. To identify ClncRNAs, Pearson and differential expression analyses were performed, resulting in 47 differentially expressed ClncRNAs meeting the criteria of |R²| >0.5 and p <0.05, and | log fold change (FC) | >1, as shown in **Supplementary Table 1**. The Sankey and volcano plots in **Figures 2A, B** depict these findings. Subsequently, the TCGA cohort was divided into training and validation cohorts in a 1:1 ratio. The clinical characteristics of each cohort are listed in **Table 1**.

**Table 1:** The clinical characteristics of patients in different cohorts.

Covariates	Total cohort	Training cohort	Test cohort	p value
Age				
<=65	296 (70.48%)	149 (70.95%)	147 (70%)	0.9148
>65	124 (29.52%)	61 (29.05%)	63 (30%)	
pN stage				
N0	341 (81.19%)	165 (78.57%)	176 (83.81%)	0.2118
N1	79 (18.81%)	45 (21.43%)	34 (16.19%)	
pT stage				
T2	143 (34.05%)	66 (31.43%)	77 (36.67%)	0.2685
T3	267 (63.57%)	137 (65.24%)	130 (61.90%)	
T4	10 (2.38%)	7 (3.33%)	3 (1.43%)	

### 3.2 Construction and validation of a prognostic ClncRNAs signature for patients with PCa

**Figure 3:**
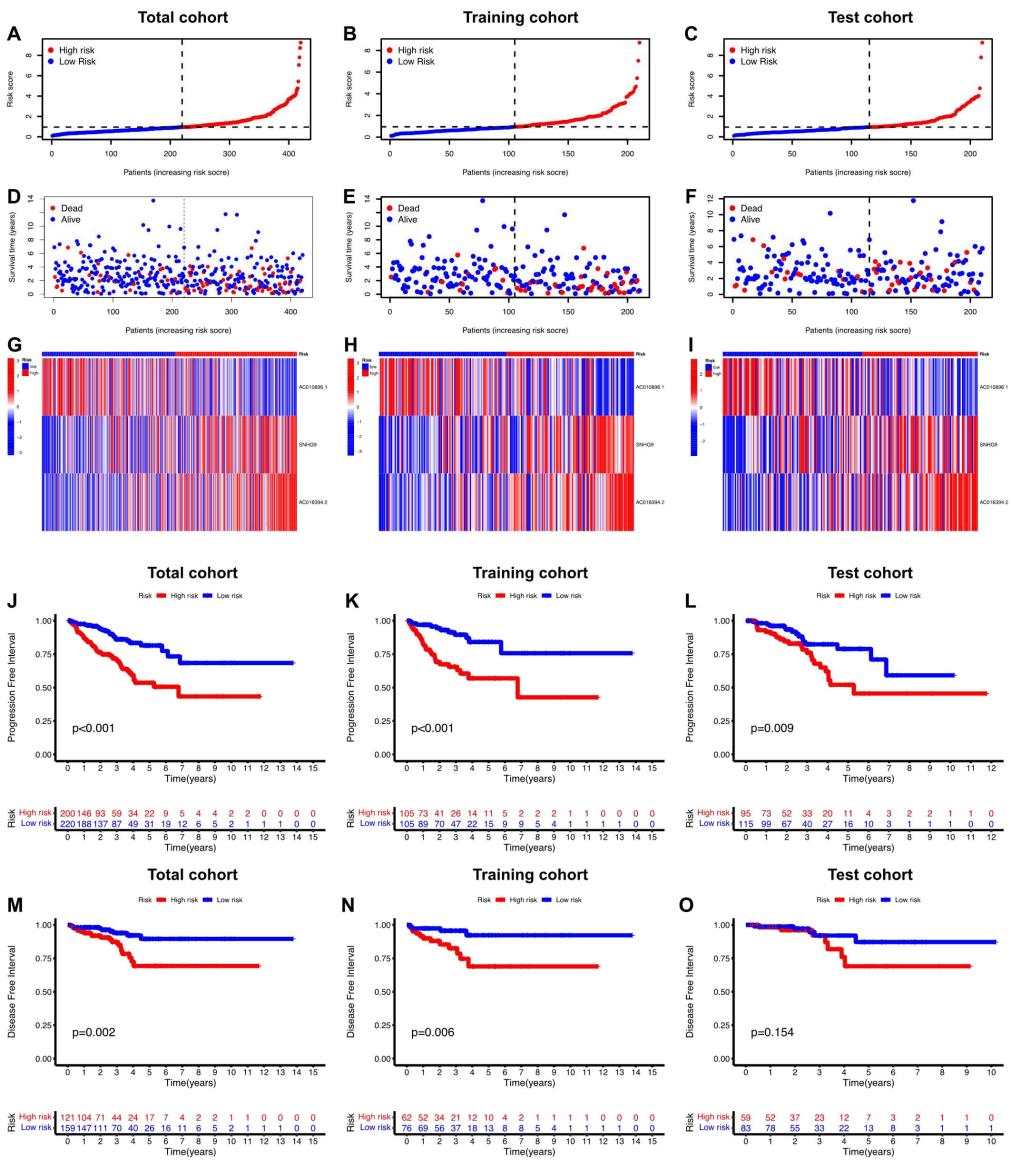
Prognosis value of the risk score of ClncRNAs signature in different cohorts. **(A-F)** The distribution of risk scores and statuses of patients with PCa in the total, training, and test cohorts, respectively. **(G-I)** Heatmaps of the three ClncRNAs expression in the total, training, and test cohorts, respectively. **(J-L)** Kaplan–Meier survival curves of PFI and **(M-O)** DFI in the high- and low-risk groups of PCa in the total, training, and test cohorts respectively.

Initially, univariate Cox regression analysis was used to preliminarily screen prognosis-related ClncRNAs, which identified six prognostic candidate ClncRNAs, including two protective lncRNAs (AC010896.1 and USP46-DT) and four high-risk lncRNAs (MHENCR, SNHG9, ACO11477.1, and AC016394.2), as shown in **Figure 2C**. Subsequently, LASSO regression analysis was conducted to refine and mitigate overfitting risks, as illustrated by the cvfit and lambda curves in **Figures 2D, E**. Multifactor Cox regression was employed to further screen for prognosis-related genes, resulting in three ClncRNAs (AC010896.1, AC016394.2 and SNHG9) with independent prognostic risk for PCa (**Table 2**). Additionally, using a heatmap, we demonstrated an association between these three ClncRNAs and 19 cuproptosis-related mRNAs (**Figure 2F**). Ultimately, based on these three ClncRNAs, a prognostic risk signature was constructed by dividing the 420 patients into high-risk and low-risk groups (1:1) using the median score as a cutoff point. The risk score for each patient was calculated with a multivariate Cox regression formula: expression level of AC010896.1*(-0.8161961) + expression level of AC016394.2*0.90231436 + expression level of SNHG9*0.44953283. Through survival status risk score curves and scatter plots for the total, training, and test cohorts, we observed a significant increase in mortality rates with increasing risk scores of the ClncRNAs signature, with the majority of deaths occurring in individuals identified as high risk (**Figures 3A–I** **).** Furthermore, the K–M survival curve analysis revealed a markedly poorer PFI in samples with high-risk scores in the total cohort (**Figure 3J**, p <0.001), training cohort (**Figure 3K**, p <0.001), and test cohort (**Figure 3L**, p = 0.009). However, when using DFI as the outcome measure, significant differences persisted between the total cohort (**Figure 3M**, p = 0.002) and the training cohort (**Figure 3N**, p = 0.06), while there was no statistical difference in the test cohort. Nonetheless, a trend of difference remained between the two groups in the test cohort (**Figure 3O**, p = 0.154).

**Table 2:** Prognostic ClncRNAs signature via multivariate Cox regression analysis.

lncRNAs	coef	HR	HR.95L	HR.95H	p value
AC010896.1	-0.8161961	0.44211019	0.20397572	0.95825825	0.03864042
AC016394.2	0.90231436	2.46530212	1.55148936	3.91734207	0.00013408
SNHG9	0.44953283	1.56757968	1.07461132	2.28669289	0.01962196

### 3.3 Evaluation of the predictive accuracy of the ClncRNAs prognostic signature

**Figure 4:**
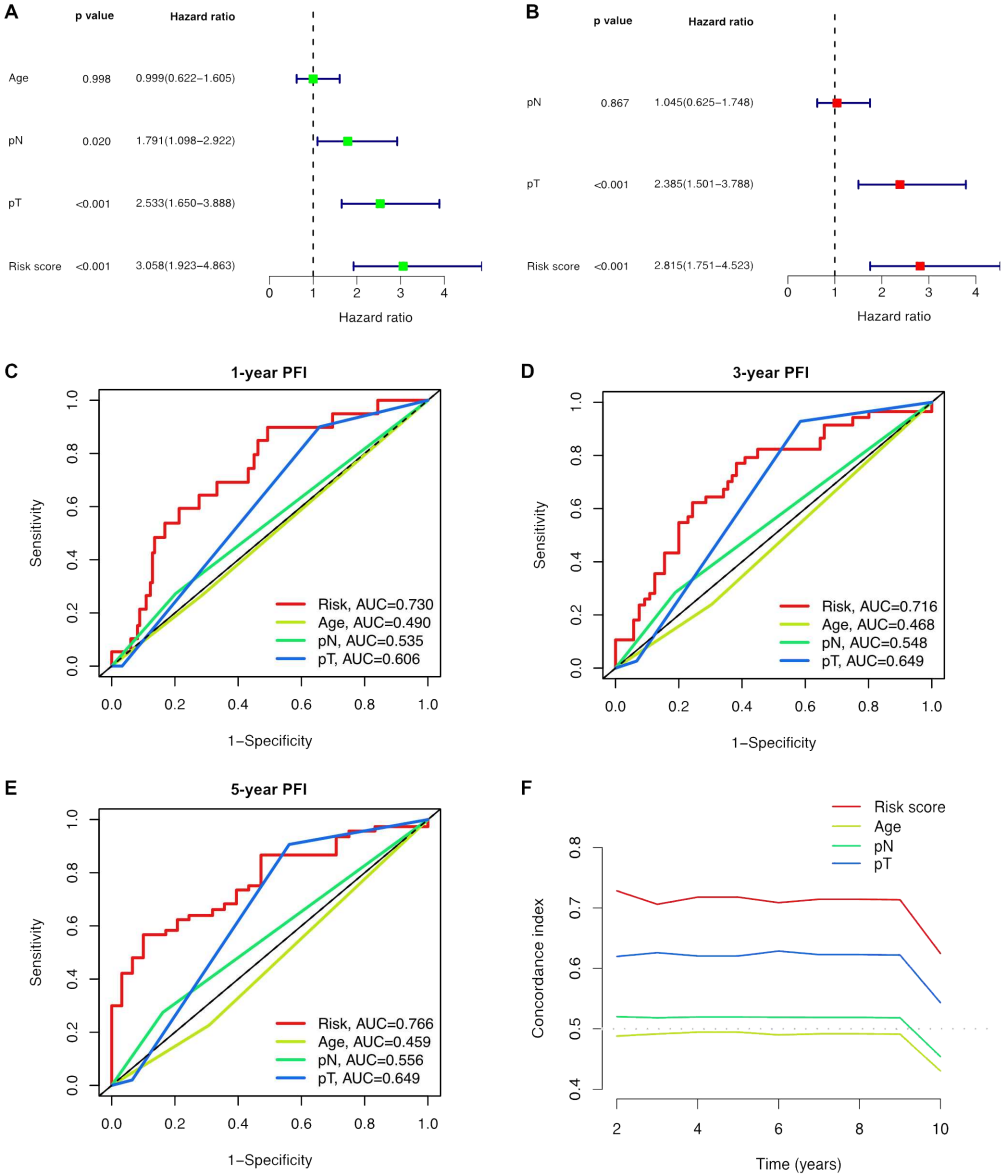
Independent prognostic analysis of the prognostic risk score of ClncRNAs signature in PCa. **(A, B)** Conducted both univariate and multivariate Cox regression analyses, taking into account the risk score and clinical characteristics. **(C-E)** TimeROC curve for 1-, 3-, and 5-year overall survival. **(F)** The C-index curve indicates that the predictive accuracy of the ClncRNAs signature risk score surpasses that of other clinical characteristics.

**Figure 5:**
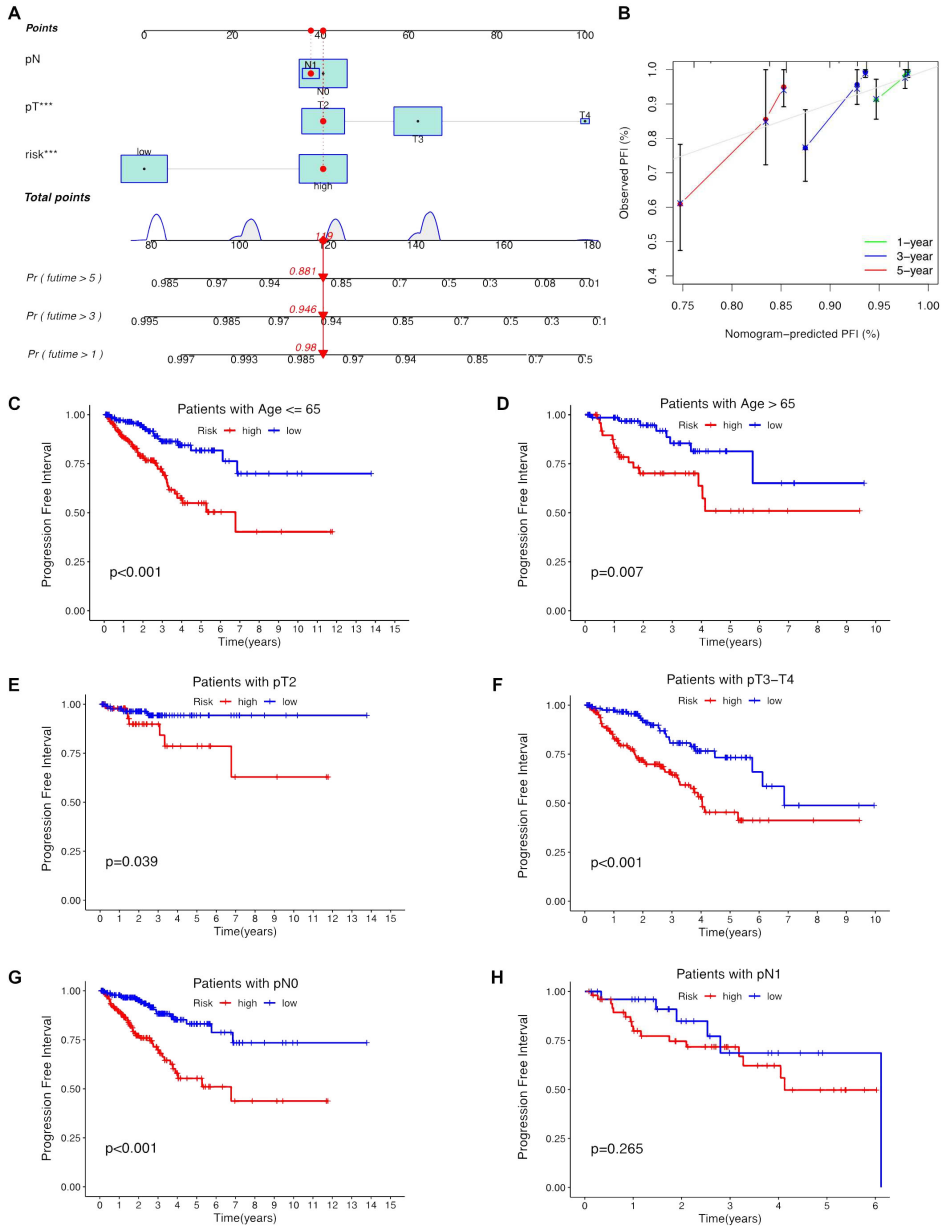
Construction the nomogram and subgroup analysis of the risk score of ClncRNAs signature. **(A, B)** The nomogram integrates the risk score and clinicopathological features to predict 1-, 3-, and 5-year DFS in PCa. **(C, D)** Kaplan–Meier curves for high-risk and low-risk groups across different age groups of patients with PCa. **(E, F)** Kaplan–Meier curves for high-risk and low-risk groups across different T stages of patients with PCa. **(G, H)** Kaplan–Meier curves for high-risk and low-risk groups across different N stages of patients with PCa.

Univariate and multivariate Cox regression analyses were conducted to validate whether the ClncRNAs signature risk score could independently predict PCa prognosis. Univariate analysis revealed significant differences in age, stage, and ClncRNAs risk score among the patients with PCa, whereas multivariate analysis excluded age as a significant factor (**Figures 4A, B**). The discriminative ability of the risk score was then evaluated against other clinical features using PFI as the outcome measure. The results showed that the ROC curve of ClncRNAs risk score had the highest AUC compared to the ROC curves of age and clinical stage, with AUCs of 0.730, 0.716, and 0.766 for the 1-, 3-, and 5-year ROCs, respectively (**Figures 4C–E**). Additionally, the 10-year C-index of the risk score was significantly higher than that of the other clinical features (**Figure 4F**). In summary, the ROC curve confirmed the significant prognostic predictive ability of the risk score of ClncRNAs signature compared with other clinical features. Furthermore, based on the results of the multivariable Cox regression analysis, a nomogram was constructed to accurately predict the 1-, 3-, and 5-year PFI (**Figures 5A, B**). To assess the accuracy of the prognostic prediction of the risk score in different stratified cohorts, a K–M survival analysis was conducted for each subgroup based on the clinicopathological characteristics including aged ≤65 years, >65 years, pT2, pT3-4, pN0, and pN1 (**Figures 5C–H**).

### 3.4 Principal components analysis and functional enrichments analysis

**Figure 6:**
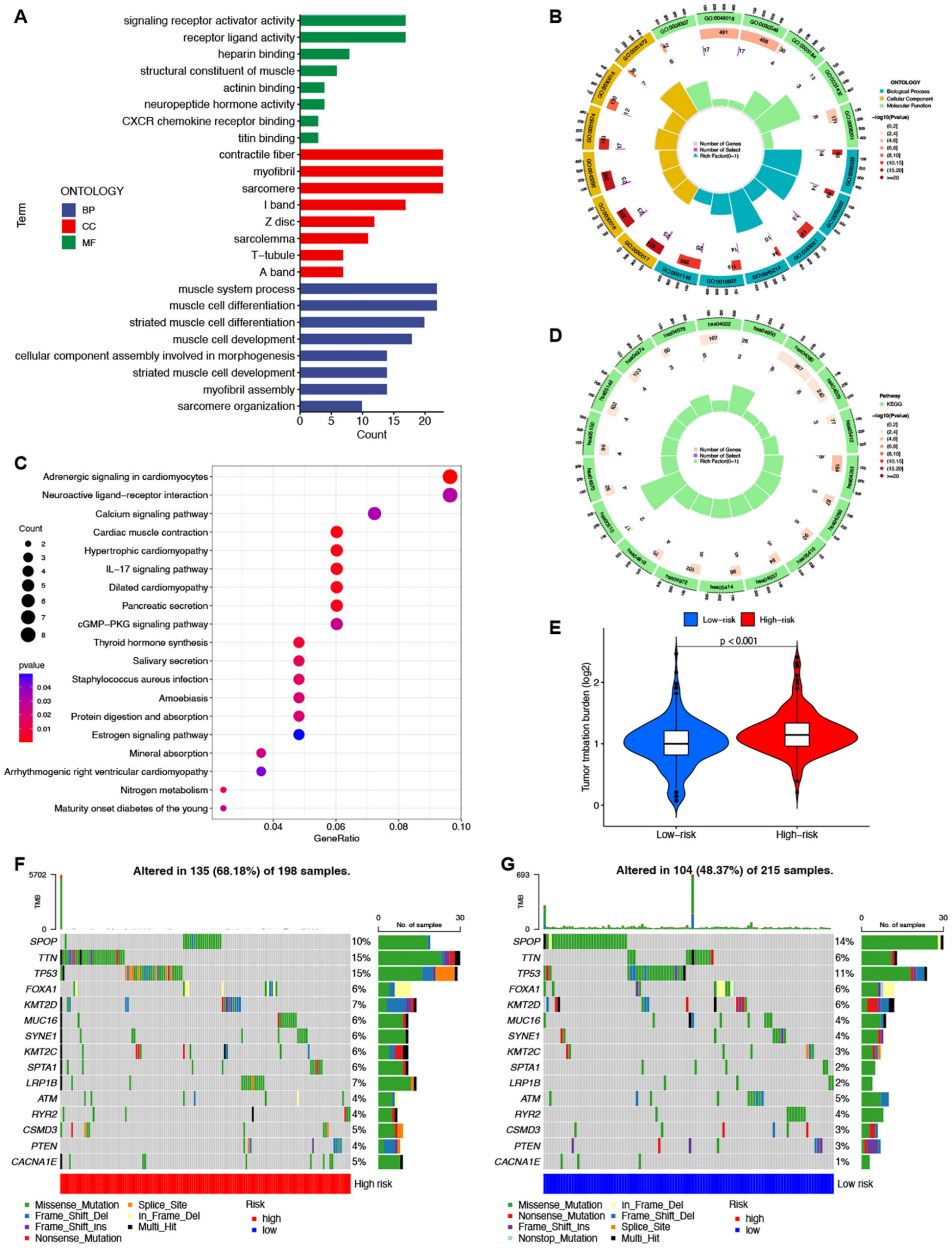
Enrichment and TMB analyses based on the prognostic ClncRNAs signature in high-risk and low-risk in patients with PCa. The GO **(A, B)** and KEGG **(C, D)** analysis shows significantly enriched biological processes and pathway between two different risk groups. **(E)** The differences of TMB scores between two different risk groups. **(F, G)** Waterfall plots showing the top 15 most frequently mutated genes in two different risk groups.

To elucidate the differences between the high-risk and low-risk groups, we conducted GO (**Figures 6A, B**) and KEGG (**Figures 6C, D**) enrichment analyses on the differentially expressed mRNAs between the two groups. GO analysis revealed potential associations of signaling receptor activation factor activity, contraction fibers, and muscle system processes with PCa. KEGG analysis indicated that enrichment was primarily in the adrenergic signaling pathway in cardiomyocytes, the neuroactive ligand-receptor interaction pathway, and the calcium signaling pathway.

### 3.5 Tumor mutation burden analysis

TMB differences between the high-risk and low-risk groups were also assessed, with the TMB of the low-risk group significantly lower than that of the high-risk group (p <0.001, **Figure 6E**). This indicates good consistency between the risk score and TMB for prognostic prediction. The waterfall plot depicted the top 15 genes with the highest mutation frequencies, highlighting the differences between the two groups. (**Figures 6F, G**). Among the top five genes with the highest mutation rates, only SPOP had a higher mutation rate in the low-risk group than in the high-risk group. The other genes showed lower mutation rates in the low-risk group.

### 3.6 Comparison of immune functions and TIDE scores in different risk groups

**Figure 7:**
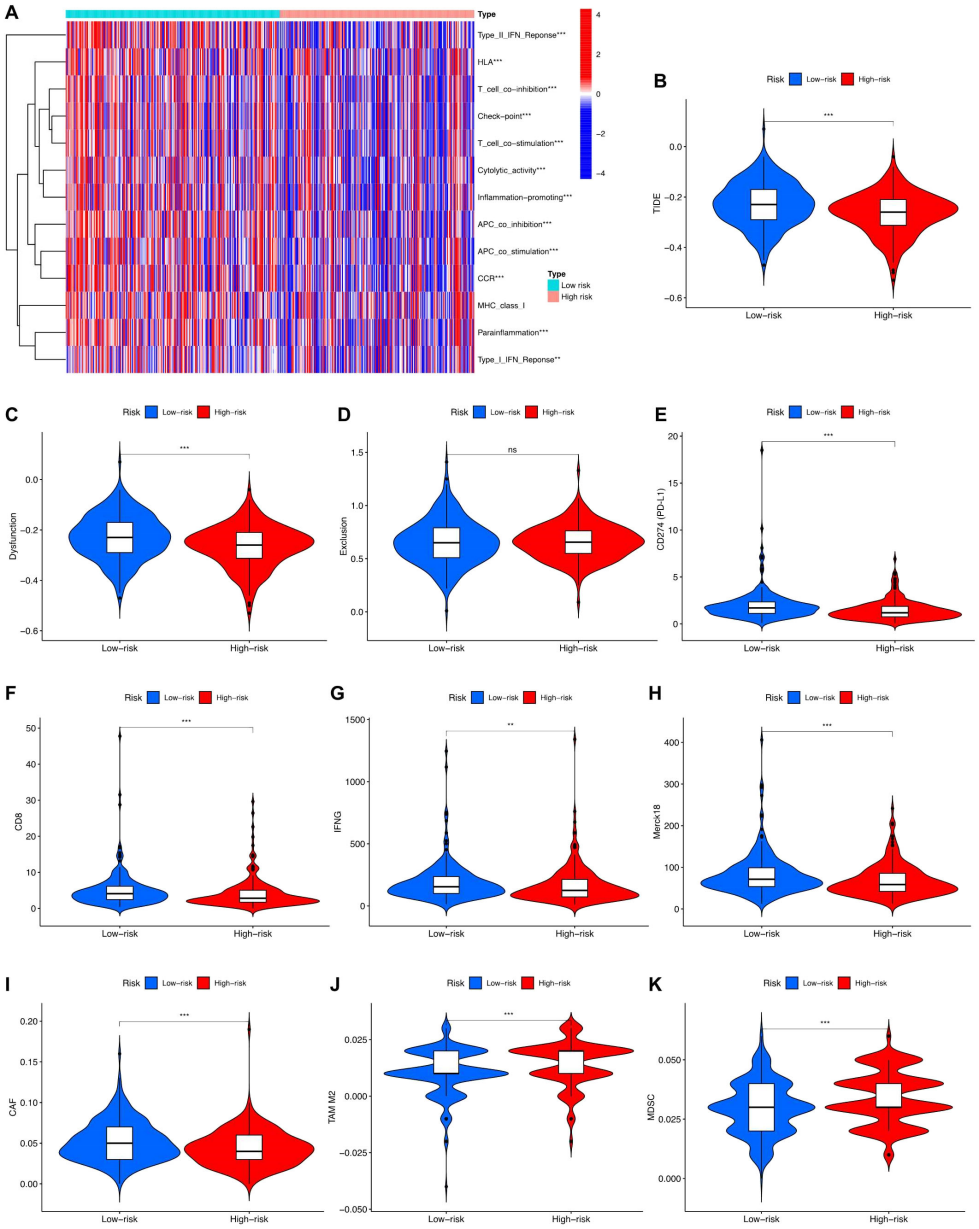
Immune function analysis in high-risk and low-risk groups. **(A)** Different proportions of immune function between the high-risk and the low-risk groups as shown in the heatmap. **(B-K)** TIDE, T cells dysfunction, T cells exclusion, CD274, CD8, INFG, Merck18, CAF, TAM M2, and MDS scores between the low-risk and high-risk groups of patients with PCa.

Through the analysis of immune-related functions, the immune statuses of the low-risk and high-risk groups were investigated. The results indicate significant differences in immune cell expression between the groups. Notably, expression levels of various immune cells, including antigen-presenting cells (APCs), co-stimulatory chemokine receptors (CCRs), pro-inflammatory markers, major histocompatibility complex (MHC) class I, sub-inflammation, and type II interferon (IFN) response, were notably lower in the high-risk group than in the low-risk group. Conversely, immune function appeared to be more robust in the low-risk group (**Figure 7A**). TIDE scores obtained from the TIDE website further supported this observation, with the low-risk group showing higher scores than the high-risk group (**Figure 7B**). Additional investigations into scores for T-cell dysfunction, T-cell exhaustion, PD-L1, CD8, IFNG, Merck18, CAF, TAM M2, and MDSC also revealed significant differences between the high-risk and low-risk groups (**Figures 7C–K**).

### 3.7 Knockdown of SNHN9 affects cell viability, migration, and proliferation in PCa cells

**Figure 8:**
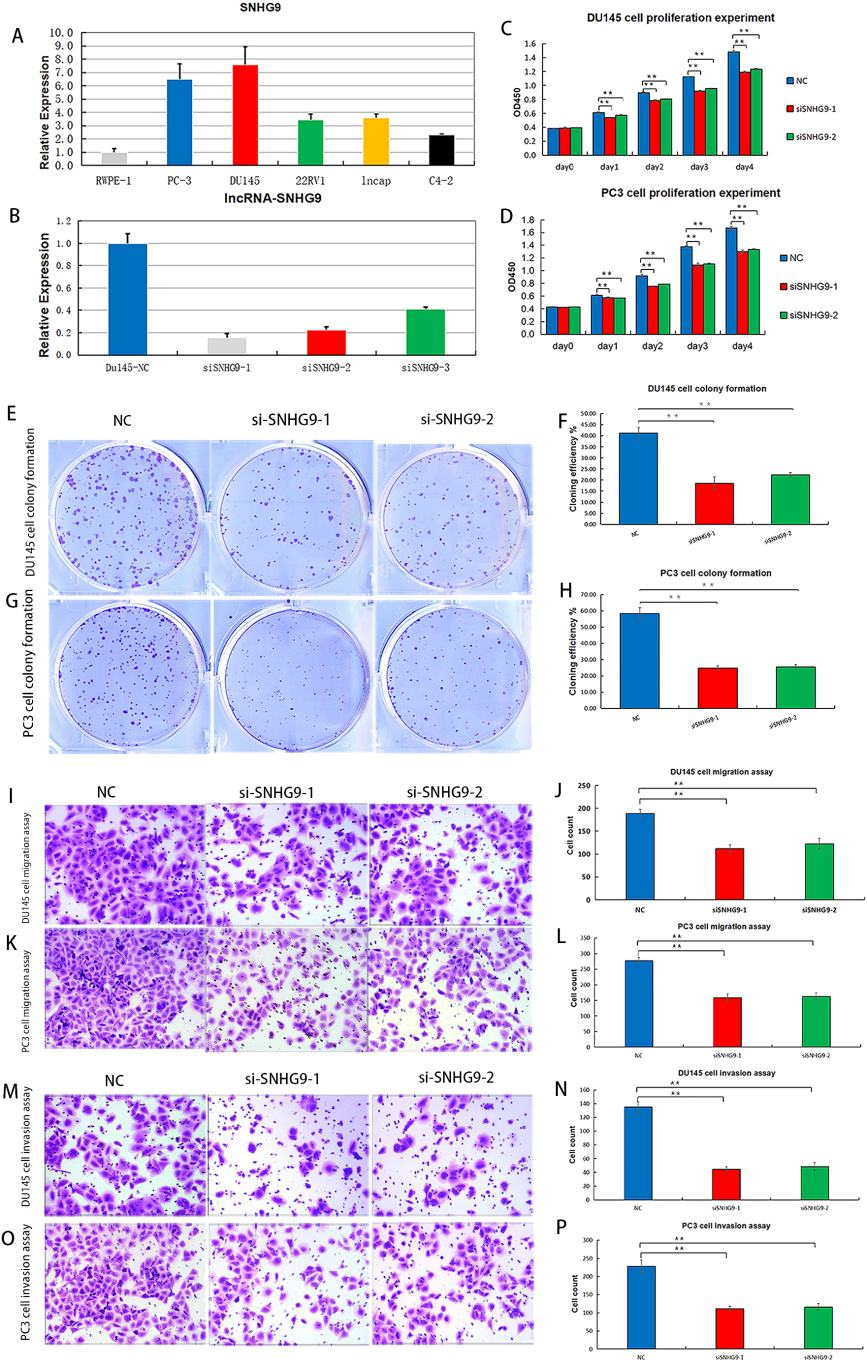
Proliferation, migration and invasion experiments after knockdown of ClncRNAs SNHG9 *in vitro*. **(A)** Expression levels of SNHG9 in human PCa cells. **(B)** Relative expression levels of DU145 cells after SNHG9 transfection with the corresponding siRNA. **(C, D)** CCK-8 assay was used to detect the effect of SNHG9 on PC3 and DU145 cells proliferation. **(E-H)** Evaluate the impact of SNHG9 on the proliferation and growth abilities of individual PC3 and DU145 cells through flat cloning experiments. **(I-L)** Use the transwell method to assess the effect of SNHG9 on the migration of PC3 and DU145 cells. **(M-P)** Use the transwell method to assess the effect of SNHG9 on the invasion of PC3 and DU145 cells. **p <0.01.

Considering that among the ClncRNAs, SNHG9 exhibited the most significantly differential expression between tumor and normal samples (**Supplementary Table 1**), it was selected for further cellular experimental validation. The expression levels of SNHG9 were measured in human PCa cells, including RWPE-1, PC-3, DU145, 22RV1, LNCAP, and C4-2 cells, using qRT-PCR (**Figure 8A**). Subsequently, functional experiments targeting SNHG9 were conducted in highly expressed PC-3 and DU145 cells. Initially, siRNA targeting SNHG9 were designed and transfected into the cells and the transfection efficiency in DU145 cells was validated by qRT-PCR (**Figure 8B**). Cell viability was assessed using a CCK-8 assay. The results indicated a significant decrease in cell proliferation in PC3 and DU145 cells after SNHG9 knockdown compared to that in the control group (**Figures 8C, D**). Colony formation assays demonstrated a marked decrease in the cloning ability of PC3 and DU145 cells after SNHG9 knockdown, with significantly fewer and smaller colonies formed, further confirming the inhibitory effect of SNHG9 knockdown on cell proliferation and growth (**Figures 8E–H**). Transwell assays revealed a significant reduction in cell migration and invasion after SNHG9 knockdown (**Figures 8I–P**).

### 3.8 Knockdown of SNHN9 affects apoptosis, cell cycle, and the expression of key cuproptosis-related mRNAs in PCa cells

**Figure 9:**
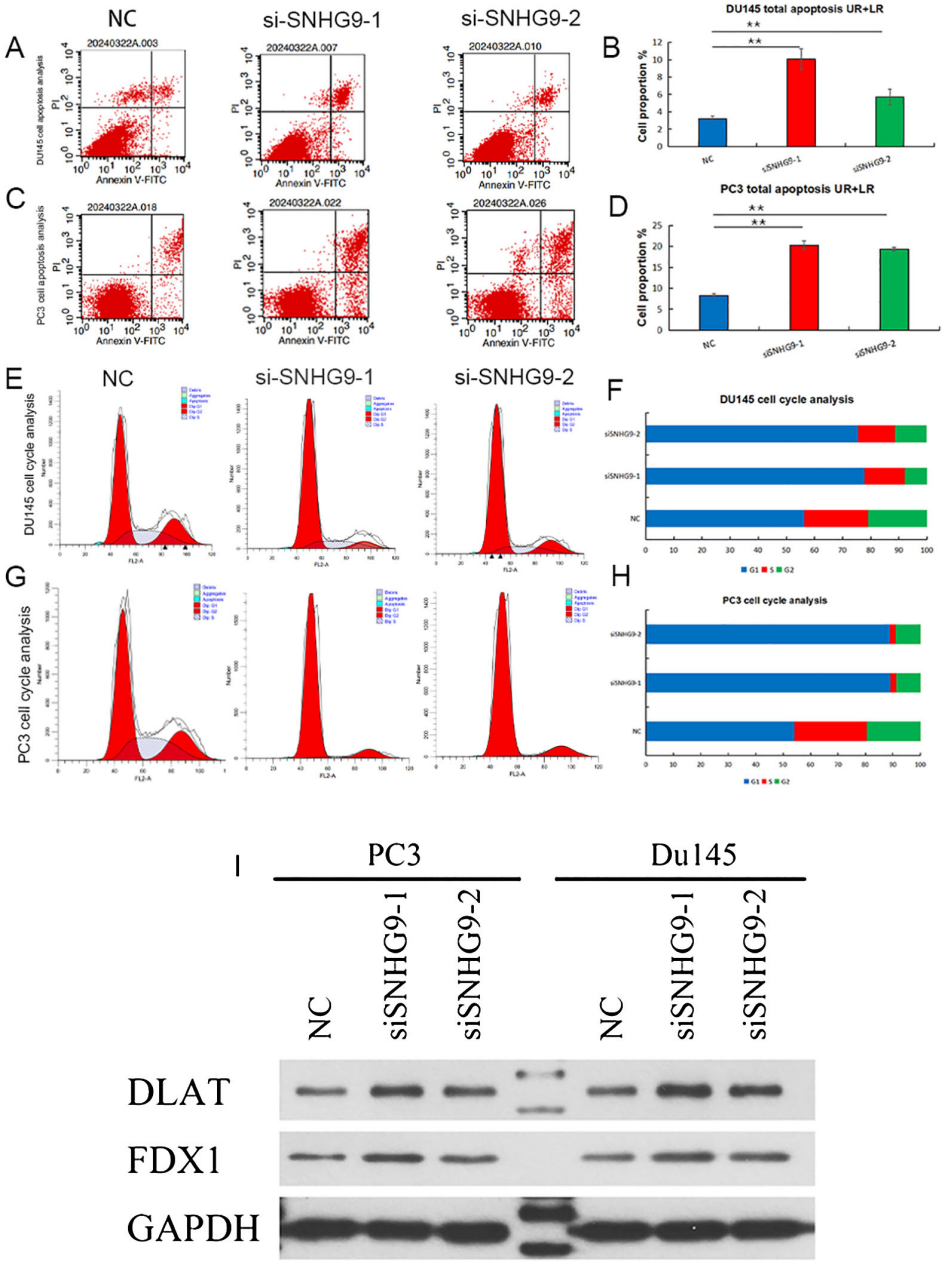
Apoptosis and cell cycle experiments, and key cuproptosis-related mRNAs validation after knockdown of ClncRNAs SNHG9 *in vitro*. **(A-H)** Examine the effect of SNHG9 on apoptosis and the cell cycle of PC3 and DU145 cells through flow cytometry. **(I)** Western blot indicated that key cuproptosis-related mRNAs (FDX1 and DLAT) were significantly upregulated in the two siSNHG9 groups relative to the control group. **p <0.01.

Flow cytometry was used to assess apoptosis and cell cycle distribution, showing increased apoptosis in PC3 and DU145 cells after SNHG9 knockdown (**Figures 9A–D**). Additionally, cell cycle analysis showed a significant increase in the proportion of PC3 and DU145 cells in the G1 phase and a decrease in the proportion of cells in the S and G2 phases after SNHG9 knockdown, suggesting inhibition of DNA synthesis and cell cycle progression (**Figures 9E–H**). Moreover, western blot analysis revealed upregulation of the key cuproptosis-related mRNAs (FDX1 and DLST) upon SNHG9 knockdown (**Figure 9I**). Collectively, these findings suggest that SNHG9 activates the proliferation, migration, and invasion of PCa cells.

## Discussion

In the study, gene expression profiles and clinical data of patients with PCa were obtained from TCGA database. Nineteen cuproptosis-related mRNAs were identified from published literature. Using Pearson’s correlation, differential expression, LASSO regression, and Cox regression analyses, three lncRNAs (AC010896.1, AC016394.2, and SNHG9) associated with prognosis were identified. These three lncRNAs are considered independent prognostic risk factors for PCa.

Previous studies have utilized these three ClncRNAs to predict the prognosis of other cancer types. For instance, the small nucleolar RNA host gene (SNHG) family comprises a group of lncRNAs that function as novel oncogenes in multiple cancers. Recent studies have shown that SNHG1 promotes immune evasion in breast cancer cells by regulating miRNA (15, 16), while SNHG6 facilitates colorectal cancer and glioma progression by modulating miR-101-3p expression (17, 18). Additionally, SNHG8 has been identified as a potential biomarker and therapeutic target for hepatocellular carcinoma and non-small cell lung cancer (19, 20). Elevated SNHG9 expression is correlated with poor prognosis, suggesting its involvement in PCa progression by influencing ribosomal function and immune cell infiltration (21). Consistently, SNHG9 was found to be the most highly expressed ClncRNAs observed in the PCa samples in our study. Functional experiments conducted on PC-3 and DU145 cell lines with elevated SNHG9 expression demonstrated significant inhibition of proliferation, migration, and invasion after SNHG9 knockdown. The expected outcomes were observed in cell cycle, clone formation, and western blotting experiments, highlighting the pivotal role of SNHG9 in PCa progression. Tu et al. (22) found that a cuproptosis-related prognostic gene signature involving AC016394.2 served as an independent risk indicator of gastric adenocarcinoma. Additionally, Kuo et al. (23) found that AC016394.2 can also serve as a lncRNA associated with disulfidptosis, distinct from other forms of cell death such as cuproptosis and ferroptosis, for prognostic prediction in gastric cancer. Therefore it is evident that these related lncRNAs play a crucial role in the regulation of tumor programmed cell death and warrant further investigation. However, the information about AC010896.1 in the published literature is limited. Therefore, further experiments are warranted in future studies to elucidate its role in PCa.

A prognostic signature was constructed based on the three ClncRNAs. To validate the predictive accuracy of risk score of the ClncRNAs signature, the survival analysis indicated the ability of ClncRNAs signature to effectively identify and differentiate between high-risk and low-risk groups. Furthermore, the risk score of the ClncRNAs signature demonstrated superior predictive efficacy for predicting 1-, 3-, and 5-year PFI compared to other clinical characteristics, with AUC values exceeding 0.70. Compared to previously published prognostic signature related to ClncRNAs, Cheng et al. (24) constructed a signature based on differentially expressed ClncRNAs, including AC005790.1, AC011472.4, AC099791.2, AC144450.1, LIPE-AS1, and STPG3-AS1. This signature demonstrated AUC values exceeding 0.70 for predicting 1-year, 3-year, and 5-year DFS. Specifically, the AUC for 5-year DFS was 0.703, while the AUC for 5-year PFI in our study reached 0.766. Additionally, Jiang et al. (25) developed a signature based on seven ClncRNAs (C1orf229, C9orf139, LIPE-AS1, MCPH1-AS1, PRR26, SGMS1-AS1, and SNHG1), which yielded an AUC of only 0.676 for 5-year DFS prediction. Although their research focused on DFS prediction, the ClncRNA signature validated through functional experiments in our study offered greater accuracy and clinical applicability. A novel nomogram was constructed to enhance the clinical applicability of the signature by combining the risk score with clinical characteristics, providing an intuitive and quantitative assessment method for predicting the 1-, 3-, and 5-year PFI in patients with PCa. Survival analysis in different subgroups revealed that the risk score of the ClncRNAs signature still exhibited highly accurate predictive capabilities for different subgroups while the predictive ability for pN1 patients was not ideal, which may be attributed to the relatively small number of pN1 patients included. Furthermore, the results of GO and KEGG analyses between high-risk and low-risk groups indicated that the differentially expressed mRNAs were mainly enriched in processes related to the muscular system and the activation and transmission of signal receptors. Previous studies have shown significant metabolic changes in myocardial cells under an increased cardiac load, with acceleration of the tricarboxylic acid cycle, leading to heightened activity in shuttle systems and biosynthetic processes (26, 27). This further confirms the intricate relationship between copper ions and various aspects of mitochondrial respiration.

The accumulation of genetic mutations is widely recognized as a primary driver of tumorigenesis (28). By analyzing the genetic mutations between the two risk groups, we found that the high-risk group exhibited a higher TMB than the low-risk group. Previous studies have indicated that in various cancer types treated with immune checkpoint inhibitors, a higher TMB correlates with better survival rates, suggesting that a higher TMB implies the presence of more mutations in tumor cells, potentially leading to the generation of more neoantigens and indicating a potentially better response to immunotherapy in high-risk patients with PCa (29). Additionally, among the top 15 mutated genes in patients with PCa, the mutation rate of TTN showed the most significant difference between the high-risk and low-risk groups, followed by SPOP and P53. Yan et al. (30) reported the prognostic value of TTN in breast cancer. Many previous studies have confirmed that SPOP and P53 mutations are among the most common mutations in PCa, with TP53 mutations indicating a poor prognosis, whereas SPOP mutations suggest the opposite. This consistency with the genetic mutation profiles distinguished by our risk scoring between the high-risk and low-risk groups further validates the predictive accuracy of risk score of the ClncRNAs signature (31, 32). The TIDE scores between high-risk and low-risk patients were also evaluated. Consistent with the findings of Zhao et al. (33), we observed that PCa patients with lower TMB and low-risk scores tended to have higher TIDE scores. Higher TIDE scores correlated with lower responsiveness to anti-PD-1 and anti-CTLA-4 therapies (34). This further supports the notion that patients with high-risk scores may respond better to immunotherapy. However, TIDE cannot predict patient survival outcomes, so this finding did not contradict the prognostic results of risk score of the ClncRNAs signature.

Currently, immunotherapy has revolutionized treatment strategies for many cancers (35); however, treatment selection for castration-resistant prostate cancer (CRPC) remains a primary challenge in PCa management. Previous study has suggested immunotherapy as a promising treatment modality for CRPC (36). However, the role of androgens in the regulation of immune function and the effects of androgen deprivation on adaptive immune responses remain unclear. Androgens are conventionally believed to possess immunosuppressive effects, and androgen deprivation has been shown to enhance T-cell function in autoimmune disease models (37, 38). Additionally, research has identified the mutation status of SPOP as an important independent prognostic marker for metastatic PCa, with SPOP mutations rendering tumor cells susceptible to androgen deprivation therapy (39, 40). In the immunocellular infiltration analysis conducted in this study, the high-risk group exhibited downregulated APC co-stimulation, C-C CCR, pro-inflammatory markers, major MHC class I, anti-inflammatory, and type II IFN response states. Previous studies have indicated that APC co-stimulation can enhance T cell activation and sustain anti-tumor immunity to improve immunotherapeutic response rates in patients with PCa (41, 42). Therefore, further investigation of the differential expression of immune cells may hold significant promise for the future treatment of PCa.

The current study had some limitations. First, our data source was singular, relying solely on TCGA database for internal validation without an external validation from additional databases. Second, while the initial validation of the identified ClncRNA SNHG9 in PCa was conducted through cell experiments, further experimental confirmation *in vivo* is required. This will be our next plan for new in-depth research projects.

In conclusion, a prognostic signature of PCa based on three ClncRNAs was established, enabling accurate prognosis prediction for PCa. SNHG9 knockdown inhibited DNA synthesis, cell cycle progression, and clonogenic ability, while upregulating cancer-related genes, suggesting its role in promoting PCa cell proliferation, migration, and invasion. This study not only deepens our understanding of the interplay between ClncRNAs and PCa but also may provide fresh insights for devising advanced therapeutic strategies aimed at enhancing the management of PCa patients.

## Data Availability

The original contributions presented in the study are included in the article/**Supplementary Material**, further inquiries can be directed to the corresponding author/s.
