# Supplementary material for: Cuproptosis-related lncRNAs emerge as a novel signature for predicting prognosis in prostate carcinoma and functional experimental validation
Source: Front Immunol. 2024 Oct 28;15:1471198. doi: 10.3389/fimmu.2024.1471198 (PMC11550951; doi:10.3389/fimmu.2024.1471198)
Supplement: Supplementary file 1 [file Table1.docx]

**Supplementary Table 1:** Differentially expressed ClncRNAs

| **lncRNAs** | **logFC** | **AveExpr** | **t** | **p value** | **PDR** |
| --- | --- | --- | --- | --- | --- |
| AC010896.1 | -1.570711834 | 1.222568897 | -15.47599914 | 3.90E-45 | 7.09E-43 |
| AC015922.2 | -24.90540532 | 19.30099421 | -13.73673066 | 3.80E-37 | 3.46E-35 |
| MAGI2-AS3 | -3.2796229 | 2.368964557 | -13.29595526 | 3.42E-35 | 2.08E-33 |
| HCG11 | -7.452712387 | 7.0115217 | -11.86455327 | 4.44E-29 | 2.02E-27 |
| ERVE-1 | -5.183543843 | 4.09455877 | -11.5580887 | 8.02E-28 | 2.92E-26 |
| LINC01679 | -2.663957132 | 1.843687703 | -11.34992389 | 5.59E-27 | 1.70E-25 |
| AC018521.6 | -3.372020712 | 4.511512839 | -9.513100149 | 5.68E-20 | 1.29E-18 |
| NNT-AS1 | -3.097642588 | 5.62223472 | -8.452639105 | 2.55E-16 | 4.09E-15 |
| LINC02693 | -1.024407186 | 1.923820434 | -8.445052829 | 2.70E-16 | 4.09E-15 |
| AL031985.3 | 2.464551248 | 4.42302821 | 7.950067937 | 1.06E-14 | 1.48E-13 |
| MHENCR | 19.88961396 | 32.85240054 | 7.028370036 | 6.19E-12 | 7.04E-11 |
| MIR22HG | -13.82946974 | 18.13132749 | -6.560699798 | 1.23E-10 | 1.25E-09 |
| AC004803.1 | -1.129888143 | 3.471102351 | -6.414771776 | 3.04E-10 | 2.91E-09 |
| FGD5-AS1 | -30.08280683 | 89.95922351 | -6.397903732 | 3.36E-10 | 3.06E-09 |
| AC022364.1 | -1.186570536 | 3.18418264 | -6.095469085 | 2.05E-09 | 1.78E-08 |
| WARS2-AS1 | 1.455445912 | 3.529707776 | 5.990359643 | 3.78E-09 | 3.13E-08 |
| SNHG9 | 30.1025638 | 51.72044105 | 5.948715812 | 4.80E-09 | 3.80E-08 |
| AC004066.1 | 2.834674133 | 4.100566727 | 5.862344273 | 7.86E-09 | 5.72E-08 |
| AC141930.1 | 9.12771682 | 10.43021049 | 5.451110753 | 7.56E-08 | 5.29E-07 |
| AC005670.3 | -1.209549559 | 3.134370163 | -5.042214421 | 6.25E-07 | 3.46E-06 |
| OIP5-AS1 | -7.74955109 | 25.80310127 | -5.041665482 | 6.27E-07 | 3.46E-06 |
| AC144450.1 | 4.789632838 | 5.355122604 | 5.041395763 | 6.28E-07 | 3.46E-06 |
| AL158212.3 | -1.565715193 | 3.464186076 | -4.990679272 | 8.08E-07 | 4.32E-06 |
| AC011477.2 | 4.231243417 | 12.64603996 | 4.951557537 | 9.80E-07 | 5.10E-06 |
| AC090559.1 | -1.11772966 | 2.073617541 | -4.793911323 | 2.11E-06 | 1.04E-05 |
| MIR200CHG | 54.89438693 | 120.5652989 | 4.685586174 | 3.52E-06 | 1.69E-05 |
| ATXN1-AS1 | -2.051838696 | 4.175049005 | -4.641921082 | 4.32E-06 | 2.01E-05 |
| AC003086.1 | 1.384708683 | 2.469050995 | 4.434113855 | 1.12E-05 | 4.95E-05 |
| AP001372.2 | -1.760425061 | 8.106598734 | -4.163536219 | 3.64E-05 | 0.000150362 |
| AL592295.6 | -2.187407639 | 10.90168373 | -4.099463962 | 4.76E-05 | 0.000184467 |
| LINC00909 | -1.282350983 | 5.802466365 | -4.094276182 | 4.87E-05 | 0.000184593 |
| AL450326.1 | -1.528992216 | 5.058182821 | -4.048184432 | 5.90E-05 | 0.000219123 |
| AC121764.1 | -1.166772728 | 0.315039964 | -4.026405292 | 6.46E-05 | 0.000234994 |
| USP46-DT | -1.990050376 | 8.494756058 | -3.881334065 | 0.000116444 | 0.000415546 |
| OTUD6B-AS1 | -1.786339087 | 9.271325316 | -3.78022092 | 0.000173761 | 0.000596689 |
| SNHG16 | 2.905121691 | 11.65278608 | 3.748287068 | 0.000196811 | 0.000663325 |
| AC011477.1 | 1.600158567 | 9.319756962 | 3.294667202 | 0.001048579 | 0.003029227 |
| AC073046.1 | 1.85102619 | 4.82620217 | 3.241204508 | 0.001262012 | 0.003480094 |
| AC016394.2 | 1.47435988 | 5.221922061 | 3.134417633 | 0.001813476 | 0.004853716 |
| CTBP1-DT | 1.893757562 | 11.38760579 | 2.997668311 | 0.002842983 | 0.007391755 |
| AC024075.1 | -3.368873476 | 9.955783363 | -2.839814793 | 0.004680416 | 0.01168107 |
| AC016747.1 | 1.779834235 | 9.438743038 | 2.828055625 | 0.004853245 | 0.011936359 |
| AL354993.2 | 1.190558909 | 3.384055696 | 2.796472025 | 0.00534647 | 0.01263711 |
| AC108673.3 | 2.195597513 | 9.385732188 | 2.742396619 | 0.006297267 | 0.014693624 |
| AC008764.2 | -4.010308909 | 17.30959259 | -2.545942921 | 0.011168964 | 0.024417949 |
| EBLN3P | 4.886515365 | 32.11950271 | 2.435641882 | 0.015180817 | 0.032126846 |
| MIR4453HG | 1.044346806 | 5.669294213 | 2.427686097 | 0.015514211 | 0.032455016 |

**Supplementary Table 2:** Primer Sequences for Quantitative Real-Time PCR (qRT-PCR) of SNHG9 and GAPDH.

| **Gene** | **Primer** | **Sequence** |
| --- | --- | --- |
| SNHG9 | Forward (SNHG9-F) | 5' GTCACCCGAAAAGCGACTAT |
|  | Reverse (SNHG9-R) | 5' GGAGGACCAGTGTCCTAAGT |
| GAPDH | Forward (GAPDH-F) | 5' GGGAAACTGTGGCGTGAT |
|  | Reverse (GAPDH-R) | 5' GAGTGGGTGTCGCTGTTGA |
