# Supplementary figures and images for: Cuproptosis-related lncRNAs emerge as a novel signature for predicting prognosis in prostate carcinoma and functional experimental validation
Source: Front Immunol. 2024 Oct 28;15:1471198. doi: 10.3389/fimmu.2024.1471198 (PMC11550951; doi:10.3389/fimmu.2024.1471198)

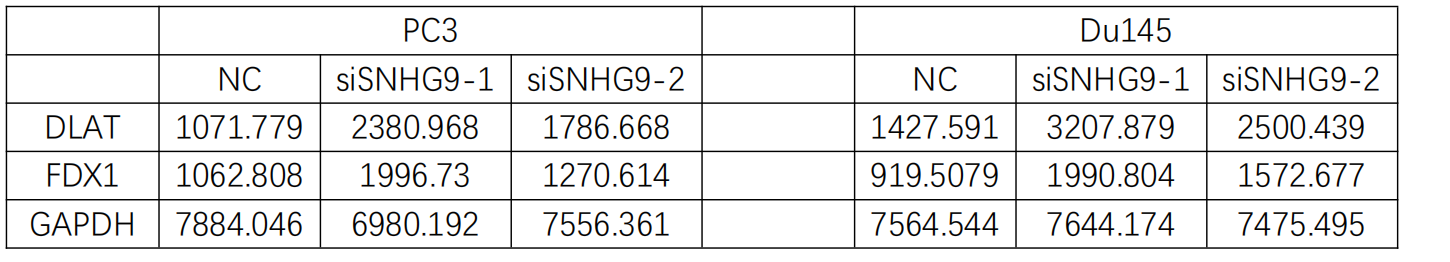


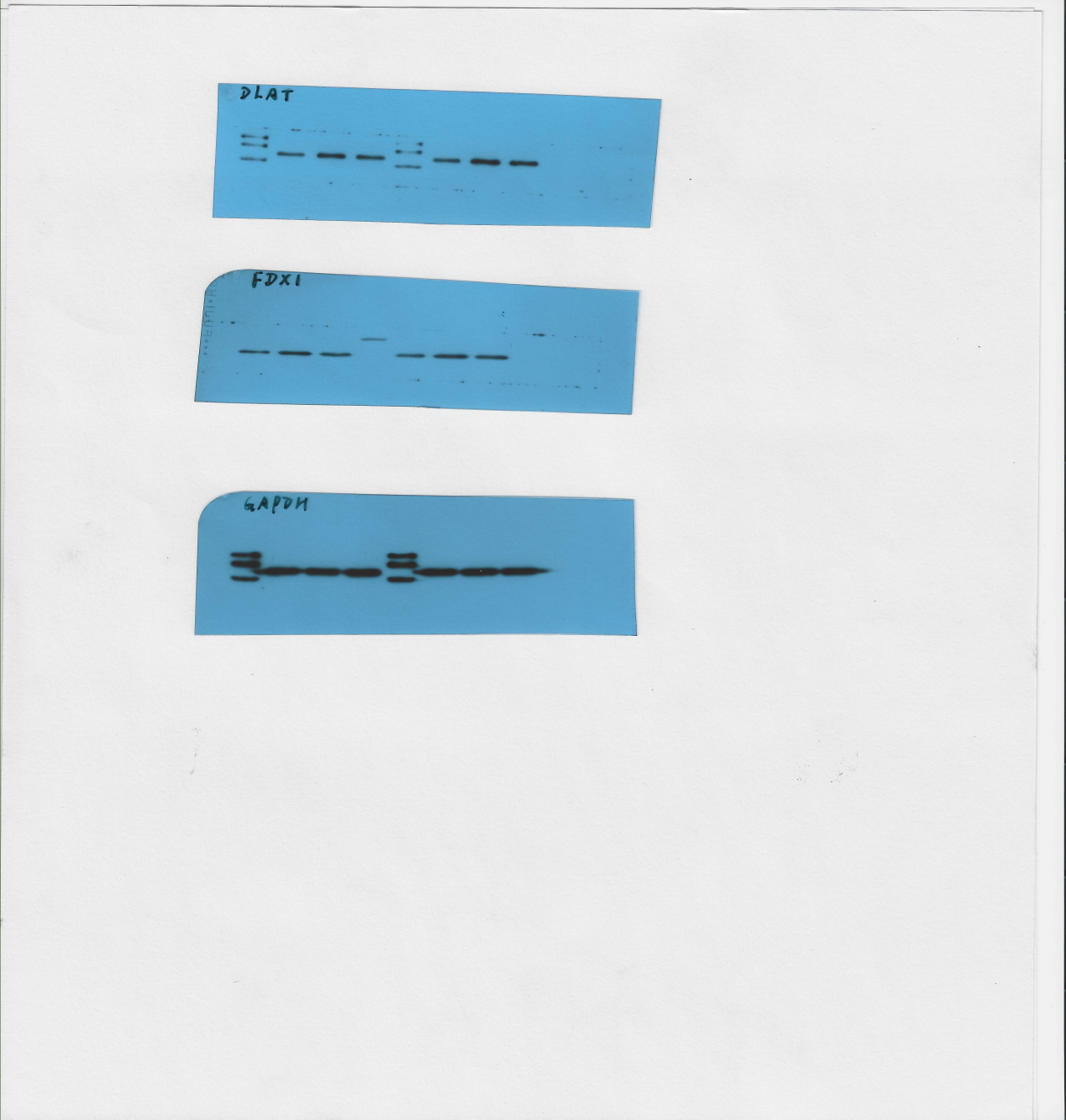

Supplement: Supplementary file 2 [file Table2.docx]
